# The Role of lncRNAs in the Stem Phenotype of Pancreatic Ductal Adenocarcinoma

**DOI:** 10.3390/ijms22126374

**Published:** 2021-06-15

**Authors:** Jorge Melendez-Zajgla, Vilma Maldonado

**Affiliations:** 1 Functional Genomics Laboratory, Instituto Nacional de Medicina Genomica, Periferico Sur 4809, Tlalpan, Mexico City 14610, Mexico; jmelendez@inmegen.gob.mx; 2 Epigenomics Laboratory, Instituto Nacional de Medicina Genomica, Periferico Sur 4809, Tlalpan, Mexico City 14610, Mexico

**Keywords:** tumor initiating cells, cancer stem cells, non-coding RNAs

## Abstract

Pancreatic ductal adenocarcinoma is one of the deadliest tumors. This neoplasia is characterized by an important cellular and phenotypic heterogeneity. In particular, it has been shown that at least two subtypes can be found: basal-like, which presents stem-like properties, and classical. Cancer stem cells have been isolated and characterized from these tumors, showing their dependance on general and tissue-specific stem transcription factors and signaling pathways. Nevertheless, little is known about their tissue microenvironment and cell non-autonomous regulators, such as long-non-coding RNAs. (lncRNAs). In this review, we summarize the current knowledge about the positive and negative effects of lncRNAs in the stemness phenotype of pancreatic ductal adenocarcinoma cancer (PDAC).

## 1. Introduction

With an overall survival of only 5% at 5 years, pancreatic ductal adenocarcinoma (PDAC) is probably the deadliest of the malignant tumors. PDAC is the eighth and ninth leading death cause by cancer in males and females, respectively, throughout the world. In addition, the number of cases and deaths due to this disease has more than doubled from 1990 to 2017, and it is expected to grow further in the next years [1]. PDAC arises from pancreatic acinar cells that lose their differentiation and convert to “duct-like” cells in a process known as acinar-to-ductal metaplasia (ADM) [2]. This process is mainly driven by a combination of *kras* mutations and tissue damage that “locks” the cells in an altered epigenetic state suitable for secondary gene mutations [3]. Thus, an important epigenetic component is present since the beginning of pancreatic cancer initiation. Several studies have shown that these initiated cells give rise to the so-called pancreatic intraepithelial neoplasia (PanIN) that progress from low grade to high grade, leading to full-blown PDAC. An alternative pathway that involves additional preneoplasic lesions, such as intraductal papillary mucinous neoplasms (IPMN), can also give rise to these tumors. As mentioned, *kras* mutations are present in over 90% of PDAC, although some reports have shown lower mutation rates for this gene [4]. In addition to *kras*, there are other recurrent gene mutations, including *tp53*, *cdkn2a, smad4, arid1a, kdm6a, robo1* and *2, slit2* and *rnf43* [4], with an additional “long tail” of mutations present at lower rates, as in the case for other tumors [5]. Nevertheless, several of the affected genes in this “long tail” belong to common pathways, including c-Myc, TGFβ, cell cycle control, WNT and NOTCH signaling, so there is an apparent convergence to the main routes involved in the genesis of these tumors [6]. PDAC is one of the most notable examples of cellular heterogeneity in cancer, given its complex composition of tumor and stromal cells. These tumors have a dense stroma consisting of pancreatic stellate cells, several extracellular matrix components, such as hyaluronic acid and type I collagen, as well as several immune cells such as macrophages, lymphocytes, plasma and mast cells, which participate in the disease progression by mean of a complex interaction between cells and micro ambient cues [7]. Furthermore, there is an additional level of intratumoral heterogeneity in each cellular component, with different subtypes of cancer-associated fibroblasts (CAF) and tumor cells. CAF are responsible characteristic dense stroma of these tumors, arising through the reprogramming of pancreatic stellate cells, mesenchymal stem cells, bone marrow-derived stem cells and endothelial cells via a reciprocal tumor-stroma network [8]. These cells are heterogeneous by origin, genotype and epigenetic phenotype [8]. In turn, pancreatic cancer cells can present at least two interchangeable phenotypes (basal-like and classical) which are determined by the tumor microenvironment (TME) [9,10], and that vary in their stem capacity [11]. A particularly interesting hypothesis is the presumption that the relative number of each of these cells in specific tumors could determine the specific PDAC subtype. There are at least two general molecular PDAC subtypes, as determined by transcriptome and genomic analyses [12] (which still present an important heterogeneity): basal-like and classical; although, additional subclassifications including progenitor subtype, squamous and aberrantly differentiated endocrine exocrine (ADEX) have also been proposed [13]. Classical tumors have *smad4* and *gata6* alterations and high expression of transcription factors related to pancreatic lineage differentiation such ad *hnf1a, gata4* and 6 and *hnf4g* [12]. Basal-like pancreatic tumors have an important imbalance in *kras* copy number and a decreased expression of lineage factors such as *gata6* [12]. These tumors, which have a stem signature, are more aggressive and present a worse prognosis [14,15]. It is important to note that basal-like PDAC present a clear dependence on the pivotal suppressor of differentiation program Enhancer of Zeste Homolog 2 (EZH2) by means of regulating the expression of the master epithelial transcription factor GATA6 [16,17]. Since it is also clear that these subtypes cannot only coexist in a particular tumor [18] but also transition between them, as revealed by single-cell sequencing [12,19], resolving the TME and cell non-autonomous signals that regulate this transition is key to fully understanding the biology of this important neoplasia.

## 2. Pancreatic Cancer Stem Cells

Cancer stem cells (CSC) or tumor initiating cells (TIC) are a functional subpopulation of cells within tumors that exhibit stem or progenitor cell properties. These cells have the ability to self-renew and give rise to a population of phenotypically diverse progeny. CSC divide either symmetrically or asymmetrically, with a limitless proliferative ability and with higher tumorigenic, invasive and metastatic potential than the rest of the tumor cells [20,21]. There are several lines of evidence that point toward the participation of these cells in the initiation and progression of several tumors [22,23]. Among these, the most relevant are lineage-tracing and transplantation studies in mice [24,25] and humans [26]. In the case of mice, tumors were created by a conditional deletion of the tumor suppressor gene Apc targeted to intestinal stem cells via *lgr5* in which individual *apc*-mutated cells were labeled red. After tumor growth, cells expressing the stem marker *lgr5* were induced to switch their color from red to blue. As expected, blue cells were able to create clonal patches, an evidence of hierarchical organization [25]. In the case of humans, CRISPR-Cas gene-editing was used to insert cassettes into the stem gene locus *lgr5* of organoids derived from colon cancer patients [26,27]. After xenotransplantation, it was found that *lgr5++* cells produced progenies that underwent progressive differentiation, and which were proportional in number to the size of the xenografts, whereas more differentiated cells produced daughter cells that remained as single cells or disappeared from the transplant.

Recent work has found that the CSC phenotype is not a fixed trait but presents with an important plasticity that is epigenetically regulated by the tumor microenvironment. For example, Gupta, et. al, found that breast cancer cell lines were heterogeneous, having distinct subpopulations with stem, basal and luminal phenotypes [28]. Each of these subpopulations was able to generate the other phenotypes, giving rise with time to the original composition of the culture in a stochastic manner. This result has been supported by recent reports in fresh human tissues [29] and in other tumors, such as ovarian [30], lung [31] and possibly pancreas [18], among others. This plasticity is mostly driven by the tumor microenvironment (TME) by a diverse number of pathways that end in epigenetic mechanisms. A clear example of this was provided by Schwitalla et al., who demonstrated that environmental Wnt activation induced dedifferentiation of non-stem cells that acquired tumor-initiating capabilities [32]. This activation is driven by NF-kappaB, a proinflammatory signaling pathway, supporting the hypothesis that tissue inflammation is an oncogenic driver. A similar result was provided by Iliopoulos et al., who showed that cytokines, in particular IL6, were the key factors that mediated the conversion of non-CSC to CSC in breast and prostate cancer [33]. In addition to cytokines, several additional TEM factors are able to regulate CSC plasticity. Hjelmeland et al. found that acidic environments similar to those found regularly in rapidly progressing tumors induce a stem phenotype in gliomas, which in turn exert paracrine effects in tumor growth [34]. All these results suggest that a supporting niche similar to their normal tissular counterpart may exist in tumors [35]. Interestingly, Lonardo, et. al., showed that pancreatic stellate cells are able to form a stem niche for pancreatic cancer cells [36]. These cells secreted Nodal, a morphogen from the TGFβ family, which acted upon CSC in a paracrine way in order to increase their numbers and invasion capabilities. Therefore, the stem phenotype in cancer is a plastic state that is dynamic in nature and is dependent on a varied combination of TME signals.

Starting with the pioneering work of Al-Hajj et al. in 2003 [37], CSCs have been identified in most of the tumors analyzed to date, including pancreatic cancer [38,39]. Li et al. were the first to report the isolation of pancreatic cancer stem cells (PCSC) using the CD24+/CD44+ ESA+ surface proteins as isolation markers [38]. Since then, markers common to other tumors have been used for the identification and isolation of these cells, including the surface proteins CD44, CD24, CD133, CXCR4, EpCAM and c-MET [38,39,40,41], as well as functional markers such as ALDH1 activity, side population and autofluorescence due to the retention of ABCG2 in the endoplasmic reticulum [42,43,44,45] (Table 1).

These cells could be derived from the initial tumoral cell clone or, as described previously, be plastic enough to be derived from more differentiated cells after specific TME signals [19]. Thus, in the later scenario, PCSC could be defined as a state more than an entity [55], which would make the elucidation of the TME determinants of this state crucial for understanding and treating these tumors [19]. These cells contribute to metastasis, chemoresistance and recurrence of pancreatic tumors and may be partially responsible for the immunosuppressive TME of these tumors [39,56,57].

Several signaling pathways are able to regulate PCSC, such as Hedgehog [58], Notch [52], WNT/Catenin [59,60,61] and JAK/STAT [53], among others [62]. These transduction signals ultimately rely on a group of stem transcription factors to regulate the cells’ phenotype, such as Myc, Oct4, Sox2 and Nanog [63,64]. PDAC are notably resistant to chemotherapy agents, showing limited benefit from gemcitabine-based schemes when diagnosed as locally advanced or metastatic disease. It has been shown that PCSC are resistant to gemcitabine [65], so these cells could be responsible for tumor progression or recurrence. The reason for this resistance is multifaceted, although an important factor is the known quiescence of these cells [66]. This quiescence is mainly sustained through cues from the TME that rely on epigenetic changes such as methylation in non-CpG islands and intergenic regions, as it has been shown that inhibition of DNA methyltransferase 1 inhibits quiescence and induces differentiation [66]. The importance of PCSC’s role in disease progression and natural history is supported by the results of Li et. al., who found that not only did CD44, a marker for PCSC, correlate with metastasis, recurrence and poor outcomes, but that an antibody directed against this surface protein was able to reduce the growth, metastasis and recurrence of human pancreatic tumor xenografts in mice [67]. Similarly, high expression levels of both CD44 and CD133 were associated with poor prognosis in a tissue microarray analysis of PDAC patients [48]. Additional examples of the potential of PCSC targeting are available. For example, Huang et al. have shown that inhibiting the Hedgehog pathway decreased self-renewal of PCSC, reversing chemoresistance to gemcitabine [58]. 

In conclusion, PCSC could be a key factor for the poor prognosis of PDAC patients. Using these cells or surrogate markers of them as diagnostic/prognostic factors or as therapeutic targets may be key to improve patients’ survival possibilities in the near future. 

## 3. Role of lncRNAs in the Stem Phenotype

**Figure 1:**
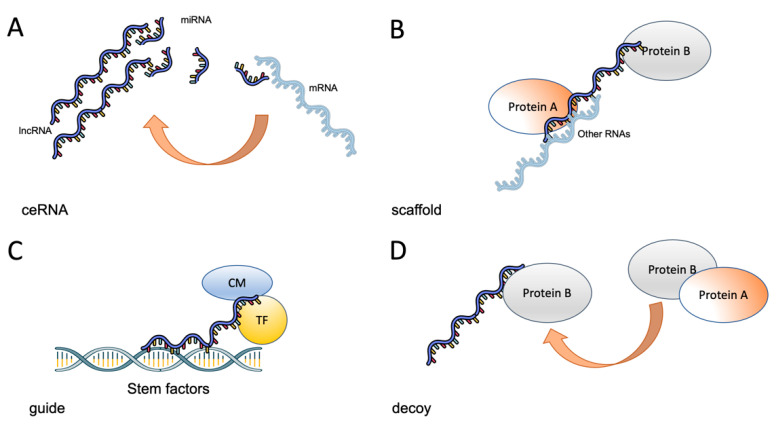
LncRNAs can be classified according to their function as: (**A**) sponges that act as competing endogenous molecules (ceRNAs) that interact with microRNAs and thus prevent them from interacting with their cognate mRNAs; (**B**) scaffold lncRNAs that act as adaptors to proteins; (**C**) enhancer or guide lncRNAs that stabilize chromosomal loops or recruit chromatin modifiers to DNA and (**D**) decoy lncRNAs, which act by sequestering proteins.

LncRNAs are a complex and heterogeneous group of transcripts, most of them without an assigned function. These RNAs can be classified by localization as sense lncRNAs that overlap coding mRNAs, antisense lncRNAs that overlap coding mRNAs but in the non-coding strand, bidirectional RNAs that share the transcription start site of other RNAs, intronic lncRNAs and intergenic lncRNAs [23]. LncRNAs can also be classified according to their function as decoy lncRNAs, which act by sequestering proteins, guide lncRNAs that recruit chromatin modifiers to DNA, scaffold lncRNAs that act as adaptors to proteins, sponges that act as competing endogenous molecules (ceRNAs) that interact with microRNAs and thus prevent them from interacting with their cognate mRNAs and enhancer lncRNAs that stabilize chromosomal loops (Figure 1) [23,68]. 

There are now numerous reports showing that these molecules are involved in the initiation and progression of a diverse array of diseases, including cancer. It has been shown that prostate cancer associated transcript 1 (PCAT-1), a lncRNA overexpressed in metastatic prostate tumors, regulates proliferation by being a transcriptional repressor for a subset of important cell-cycle genes [69]. Similarly, the lncRNA ANRIL (antisense non-coding RNA in the INK4 locus) interacts with SUZ12, part of the PRC2 complex, to repress the expression of the tumor suppressor gene *p15* (*ink4b*), thus increasing proliferation [70]. LncRNAs can also participate in invasion and metastasis, as shown by the reported actions of MALAT1 (metastasis-associated lung adenocarcinoma transcript-1), which is able to regulate several cellular functions associated with these processes, such as migration, apoptosis, gene expression, etc [71]. In addition, a role of lncRNAs in modulating the TME dynamics has been found. In particular, it has been shown that several lncRNAs regulate angiogenesis [72]. For example, maternally expressed gene 3 (MEG3) is an imprinted gene that expresses a lncRNA that is frequently downregulated in tumors. This RNA functions as a tumor suppressor gene (TSG) that represses angiogenesis [73]. Adding to this, it has been also found that lncRNAs are able to regulate distinct forms of cell death, such as ferroptosis, apoptosis and autophagy. For example, Li, et. al., found that lncMIF-AS1, a lncRNA widely upregulated in several tumors, was able to upregulate the expression of the cytochrome oxidase subunit NDUFA4 in order to decrease apoptosis and increase proliferation [74]. This effect was mediated by a competing endogenous RNA (ceRNA) mechanism in which miR-212-5p is “sequestered”, precluding its binding to NDUFA4 mRNA (see Figure 1). Similarly, it has been found that LINC00336 is able to inhibit ferroptosis in lung cancer cells [75]. This is achieved by a ceRNA mechanism involving miR-6852 and the protein cystathionine-b-synthase, a surrogate marker of ferroptosis. As expected, from these results, lncRNAs are also able to regulate the response to chemotherapy. In addition to the mentioned effects on cell death, lncRNAs regulate drug efflux pumps, cell cycle and DNA repair mechanisms, all processes important to chemo and radiotherapy responses. For example, Wang, et. al, showed that the lncRNA UCA1 (urothelial carcinoma associated 1) de-repressed the drug pump ABCB1 through a ceRNA mechanism involving miR-129, making ovarian cancer cells chemoresistant to paclitaxel [76]. DNA repair can also be regulated by lnRNAs, as demonstrated by Wu et al., who showed that lnc-TALC increased the expression of the DNA repair enzyme O6-methylguanine-DNA methyltransferase (MGMT), increasing chemoresistance to temozolomide in glioblastoma tumors [77]. Thus, lncRNAs are able to regulate most if not all cancer hallmarks [78] by fine-tuning basic cellular networks and relying on contextual signals in the TME.

It has been recently shown that lncRNAs are important regulators of the stem phenotype in several tumors, including breast, esophagus, prostate, lung, colon, liver, kidney, stomach, bone and liver cancers [23]. As key molecules that regulate a plethora of processes through epigenetic and transcriptional mechanisms, they are in the right place to modulate differentiation phenotypes that require a relatively quick and plastic response. There are several examples of the role of lncRNAs on the stem phenotype regulation and the list is continuously growing. The effects of these RNAs are varied and include intranuclear and cytoplasmic actions, such as a acting as a scaffold for chromatin proteins [79], competing endogenous RNA toward specific microRNAs (ceRNAs) (Figure 1) and mRNA stability regulation [80]. For example, in our group, we were able to show that a novel lncRNA (lncRNA-HAL) promoted stemness in breast cancer cells by an epigenetic mechanism, acting as a scaffold [81]. Even with all the recent publications, there are still large voids in our knowledge and there is an important need to assess the importance of lncRNAs on specific tumors stem cells, since it has been shown that lncRNAs act in a tissue-specific manner [82].

Gene expression analysis has shown that lncRNAs can be assigned and/or used as a basis for cancer classification into subtypes [83,84]. Since the first report of Du et al. in 2013 [85], several additional studies have been published that further support these findings. Most of these classification efforts derive useful prognostic information. For example, in colorectal cancer, six lncRNAs can be used as a signature for disease-free survival, classifying tumors into high- and low-risk categories [86]. Similarly, in breast cancer patients, a lncRNA classification system was able to identify four clusters that correlated with the well-known PAM50 classification, which already has a strong predictive utility [87,88]. Another example is glioma, in which a consensus clustering of lncRNAs showed the presence of three molecular subtypes: lncRN1, lncRN2 and lncRN3, with clear prognostic utility [89]. In clear-cell renal carcinoma, an analysis of 475 primary tumor samples showed that these tumors can be classified into four subtypes, C1–C4, with worse prognosis for the C2 subgroup [90]. In endometrial carcinoma, a similar study using unsupervised clustering of 1931 lncRNAs classified tumors in three subtypes: basal-like, luminal-like and CTNNB1-enriched, with basal-like having a worse prognosis [83]. This strategy has been successfully applied in novel ways. Li et al. recently created an algorithm, lmmLnc, aimed at identifying lncRNA regulators of immune-related pathways. With this tool, these authors were able to identify three molecular subtypes (proliferative, intermediate and immunological) of non-small cell lung cancer [91]. These subtypes differ in the immune cell infiltration and should be useful as predictive tools for these patients. Moreover, specific lncRNAs signatures or individual lncRNAs can be used to provide predictive information in specific clinical scenarios. For example, a panel of seven lncRNAs aberrantly expressed in paclitaxel resistant ovarian cancer tissues was used as a surrogate with high predictive value of chemoresistance in these patients, and with a secondary ability to associate with worse progression-free survival [92]. Similarly, the lncRNA MFI2-AS1 was identified as a strong predictor of recurrence in sporadic localized clear-cell carcinoma [93]. The use of this lncRNA improved patients´ stratification and, in particular, was a good predictor for increased relapse risk [93]. Similarly, a signature of eight autophagy-related lncRNAs was helpful in providing postoperative risk stratification in clear-cell carcinomas [94]. lncRNAs can also be used as a refining predictor, as exemplified for breast cancer, in which a group of 210 lncRNAs can not only be used to produce a four-subtype classification but can also provide important correlations between dependence pathways, such as PI3K in luminal A tumors and basal-like lncRNAs, and the activation of EGFR-dependent pathways [95]. In addition to these observations that show the possibilities of measuring lncRNAs’ expression for tumor subclassification, lncRNAs expression levels can be used to classify multiple cancer types. A recent example of this is the work of Al Mamun et al. that employs a deep neural network platform to show that the expression of these transcripts is able to be distinguished not only between tumor subtypes but also among tumor types [96]. A recent study explored the subtype-specific expression of lncRNAs in classical and basal-like subtypes in pancreatic cancer [97]. These authors found that 27 deregulated lncRNAs have a significant different expression pattern in these subtypes, suggesting context-dependent roles for them. Among these, the strongest correlation was found with the basal-like PDAC subtype.

In conclusion, there is ample evidence that lncRNAs are key to most of the molecular processes required for determining the stem phenotype and are uniquely positioned for fine tuning this identity to provide a crosstalk with the TME.

## 4. Role of lncRNAs in Pancreatic Cancer Stemness

As in other tumors, lncRNAs play an important role in the initiation and progression of PDAC. This role is mediated by basic cellular processes such as proliferation, cell death, invasion, metastasis and angiogenesis. Although most of the research conducted to date has explored common oncogenic lncRNAs, there is undoubtedly a role for tissue-specific lncRNAs in pancreatic carcinogenesis. HOTAIR is a 2158-bp lncRNA whose gene is located in the developmental HOXC gene cluster. This RNA binds to polycomb repressive complex 2 (PRC2), which gives it the potential to regulate several differentiation processes. The expression of this lncRNA is increased in PDAC with the highest levels in more advanced disease [98]. HOTAIR regulate invasiveness and apoptosis in PDAC cell lines by a mechanism that involves both PRC2-dependent and -independent mechanisms [98]. As mentioned before, MALAT1 is a conserved oncogenic lncRNA which is upregulated in PDAC patients. This RNA enhances proliferation, migration and invasion in pancreatic cancer cells [99], so it is a prognostic factor for worse outcomes for these patients [100] and an independent predictor of disease-free survival [101]. MALAT1 promotes proliferation and metastasis by stimulating autophagy [102]. It also regulates migration [103] and invasion by a non-PRC2 pathway that involves it acting as a ceRNA [104], regulates apoptosis and modulates the stem phenotype in pancreatic cancer cells (see below). H19 is an imprinted gene that is upregulated in PDAC. This lncRNA antagonize let-7 (an oncogenic microRNA) promotes epithelial–mesenchymal transition and metastasis [105]. Interestingly, the promoter of this gene is being explored as a novel therapy by fusing it to the diphteria-toxin gene in a plasmid in order to target pancreatic tumors [106]. lncRNA can also act as tumor suppressor of PDAC. For example, growth arrest-specific transcript 5 (GAS5) is able to inhibit the proliferation, survival and invasion of PDAC cells [107,108]. This effect is mediated by a blockage of the cell cycle by the inhibition of cyclin-dependent kinase 6 (CDK6). These are only a few examples of the expanding discoveries showing that lncRNAs are key molecules involved in most of the cellular and molecular processes required for the initiation and progression of PDAC. Recent studies have shown that the stem phenotype of pancreatic cancer cells is also a process that is regulated by these RNAs. Most of the reports analyzing the effect of lncRNAs in PCSC show that common stem cell transcription factors can be a direct or indirect target for these RNAs (Table 2). These molecules can act in a cell-autonomous and non-autonomous fashion to modulate the stem phenotype in PCSC.

**Figure 2:**
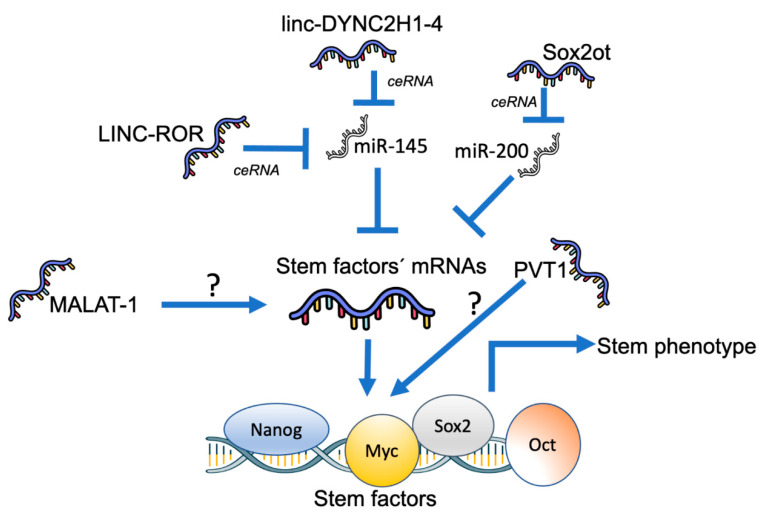
Several lncRNAs modulate the stem phenotype of pancreatic cancer stem cells by a direct effect on stem factors such as Nanog, Myc, Sox2, Oct4, etc. The most common molecular mechanism for this effect is acting as a competing endogenous RNA toward specific microRNAs. There have been hundreds of ceRNA reported examples since the initial description of this mechanism [118]. In this scenario, a microRNA that downregulates a group of mRNAs is “sequestered” by a pseudogene or a lncRNA in order to decrease its activity. This effect is thus a way to create a large-scale regulatory network.

Since it has been demonstrated that PCSC number and function depend on these factors [63], these works underline the importance of stem lncRNAs in the maintenance of the stem phenotype in PDAC (Figure 2).

The first report, to our knowledge, was produced in 2015 by Jiao et al. [109]. These authors found that MALAT-1, a known oncogenic lncRNA, was able to increase the number of pancreatic CSCs which lead to increased tumorigenicity in vivo. Although not fully explored, this effect was possibly due through regulation of the stem cell transcription factor Sox2 [109]. In 2016, Gao and collaborators found that the lncRNA LINC-ROR (long intergenic non-protein coding RNA-regulator of reprogramming) was able to act as a competing endogenous RNA (ceRNA) toward miR-145, activating the de-repression of the stem transcription factor Nanog, and thus decreasing the tumorigenic ability of pancreatic cancer stem cells (as defined by a CD24+/cD44+/ CD133+ phenotype) [110] (Table 2). This result parallels the known sponge effects of LINC-ROR in human embryonic stem cells [119]. *hoxa9* is a homeobox gene that regulates the key stem genes in hematopoietic cells [120]. Therefore, it was not unexpected that the lncRNA HOTTIP was able to regulate its expression by binding to the protein Wdr5 [59]. This interaction increases HOXA9 expression and enhances the stemness phenotype through the Wnt pathway. Interestingly, it has been shown that the Wnt signaling pathway is key to the stemness phenotype in pancreatic cancer, being associated with poor prognosis in these patients [60]. Similarly, the lncRNA Sox2ot regulates PCSC by competitively binding to miR-200, acting as a ceRNA towards Sox2 mRNA [114] (Table 2). Interestingly, this lncRNA acts in a cell non-autonomous fashion, as it is contained within secreted exosomes, once again showing the importance of the TME in PDAC progression. A more comprehensive regulation of stem factors in pancreatic cancer was found for linc-DYNC2H1-4, which exerted a positive effect on the regulation of ZEB1, Nanog, Sox2 and Oct by means of acting as a ceRNA of miR-145 [112]. Finally, a recent article by Yoshida et al. showed that PVT1 is able to regulate c-Myc in the EZH2-PVT1-c-Myc axis [113]. It is important to note that the expression of at least three of these lncRNAs is able to predict the efficacy of the main chemotherapeutic agent for pancreatic cancer, gemcitabine [121]. This finding not only underlines the importance of lncRNAs in the stemness phenotype of pancreatic cancer cells but points toward the possibility of creating a dedicated genetic firm with clinical relevance. 

**Figure 3:**
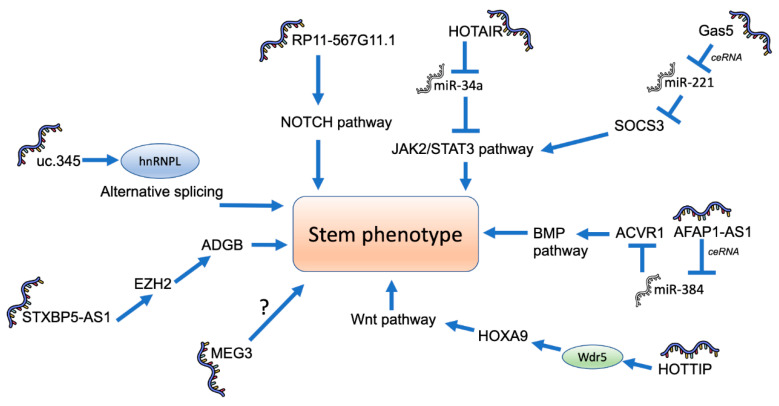
Several lncRNAs modulate the stem phenotype of pancreatic cancer stem cells by an indirect effect on stem factors such as Nanog, Myc, Sox2, Oct4, etc. This indirect mechanism involves various signaling pathways previously associated with stemness. Nevertheless, most of final stages that connect these pathways with the stem factors are currently unknown.

In addition to the direct modulation of stemness transcriptional factors, it has been shown that several lncRNAs are able to regulate PCSC in an indirect way (Figure 3). 

In 2016, Liu et al. found that the lncRNA uc.345 was able to increase the number of CD44+/CD24+ cells and Oct4, Sox2, Nanog and CD133 levels in pancreatic cancer cells by regulating the levels of hnRNPL, an important splicing factor [111]. Since it has been shown that splicing is a key process required for the stem phenotypic switch that cancer cells present [122], the participation of lncRNAs is not unexpected. Nevertheless, almost nothing is known about the molecular mechanistic aspects of their role in splicing regulation. Recently, it has been reported that the lncRNA GAS5, a reported tumor suppressor, functions as a ceRNA for miR-221, and thus regulates the expression of the suppressor of cytokine signaling 3 (SOCS3) protein [115]. This regulation inhibited the expression of Oct4, CD133, Nanog and Sox2. Similarly, MEG3, an imprinted long non-coding RNA with tumor suppressor properties, decreased the number of CD44+/CD24+/ESA+ pancreatic cancer cells and the levels of Nanog and Oct4 expression [116]. The Notch pathway is an important regulator of stemness in pancreatic cancer, since its inhibition leads to an important decrease in the number of PCSC [123]. It has been recently shown that the lncRNA RP11-567G11.1, which is upregulated in in poorly differentiated pancreatic tumors, increases the number of PCSC, probably by activating the NOTCH signaling pathway [50]. AFAP1-AS1 is an additional lncRNA that acts as a ceRNA for miR-284 to increase the expression of stem factors Oct4, ABCG2, Nestin, CK19 and CD133 by means of upregulating the BMP signaling protein ACVR1 [117]. More recently, Chen et al. showed that STXBP5-AS1 is able to recruit EZH2, a Polycomb-group (PcG) family member to the promoter of Androglobin, in order to regulate the expression of Sox2 and Nanog and the number of PCSC [15]. Finally, it has been shown that HOTAIR regulates the JAK2/STAT3 signaling acting as a ceRNA for miR-34a in order to increase the expression of PCSC markers [53,54]. 

Even with all these examples, we envision that there are several lncRNAs that regulate the stemness phenotype of PCSC that have yet to be discovered. For example, there are several lncRNAs that have been shown to participate in pancreatic cancer progression through signaling cascades that are associated with stemness but have not been validated. For example, a recent article by Yang et al. [124] showed the differential expression of several hundred lncRNAs in a 3D model used for the enrichment of PCSC. Similarly, Liu et al. identified 1503 lncRNAs that were regulated by the Wnt signaling cascade and, using the CRISPRi platform, showed that 13 were found to modulate cell growth [61].

Finally, a remaining question is related to the direct or indirect regulation of the master transcription factor GATA6 by lncRNAS, since recent works have shown that the stem phenotype of PDAC depends on the downregulation of this factor [16,17,125].

## 5. Conclusions

LncRNAs are a heterogeneous group of transcripts with complex functions that are just being uncovered. Since these non-coding RNAs show a remarkable tissue-specific expression and the ability to modulate basic genomic mechanisms, it is not unexpected that they may play an important role in the maintenance and regulation of stemness in cancer. The role of several new lncRNAs on pancreatic cancer stem phenotypes have been discovered, and more of them will surely be uncovered in the coming years. We expect that these RNAs will be useful for prognostic, if not therapeutic, targets for this deadly disease.

## Figures and Tables

**Table 1:** Pancreatic cancer stem cells markers.

Marker	References
ABCG2/Autoflourescence	[45,46]
ALDH1	[47]
CD44/CD133	[48]
CD44/CD24/EpCAM	[38]
CD133/CXCR4	[39,49]
CD133	[46]
CXCR4	[49,50]
CD44/c-Met	[40]
Nestin	[51]
Notch2	[52]
Side population	[44,53,54]

**Table 2:** lncRNAs involved in PDAC stem phenotype.

lncRNA	Effect on Stemness	Cellular Mechanism	Molecular Mechanism	References
MALAT-1	Positive	SOX2 regulation	Unknown	[99,109]
ROR	Positive	Nanog regulation via ceRNA of miR-145	ceRNA	[110]
c.345	Positive	hnRNPL regulation	Unknown	[111]
HOTTIP	Positive	HOXA9 regulation via Wdr5 binding	Protein binding	[59]
linc-DYNC2H1-4	Positive	Regulation of ZEB1, Nanog, Sox2 and Oct via ceRNA of miR-145	ceRNA	[112]
PVT1	Positive	Regulation of c-Myc	Unknown	[113]
Sox2ot	Positive	Regulation of Sox2 via ceRNA of miR-200	ceRNA	[114]
GAS5	Negative	Regulation of SOCS3 via ceRNA of miR-221	ceRNA	[115]
MEG3	Negative	Unknown	Unknown	[116]
RP11-567G11.1	Positive	Regulation of NOTCH signaling pathway	Unknown	[50]
AFAP1-AS1	Positive	Regulation of ACVR1 via ceRNA of miR-384	ceRNA	[117]
STXBP5-AS1	Negative	Regulation of EZH2 activity on ADGB transcription	Protein binding	[15]
HOTAIR	Positive	Regulation of JAK2/STAT3 signaling via ceRNA of miR-34a	ceRNA	[53,54]
